# Ethical and Clinical Challenges in Involuntary Hospitalization for First-Break Psychosis

**DOI:** 10.7759/cureus.75462

**Published:** 2024-12-10

**Authors:** Eric Chen, Justin N Passman, Ilana Yel, Mason Chacko

**Affiliations:** 1 Psychiatry, Renaissance School of Medicine at Stony Brook University, Stony Brook, USA

**Keywords:** bioethics, clinical decision making, c-l psychiatry, first-break psychosis, involuntary hospitalization

## Abstract

In our report, we discuss the case of a young adult female who presented to our institution’s emergency department with new-onset third-degree heart block and psychotic-like symptoms. The patient had a psychiatric history remarkable for presumed bipolar disorder, anxiety, depression, and cannabis use disorder, with no inpatient admissions or suicide attempts and not taking any psychotropic medications. While in our care, the patient expressed grandiose delusions and hallucinations. All laboratory and diagnostic testing (including tick-borne diseases) were within the normal limits. The patient did not meet strict criteria for involuntary hospitalization in New York State, but the care team faced a dilemma about the patient’s safety and fitness for discharge. Here, we discuss this commonly encountered scenario and the bioethics and clinical decision-making to ensure the presenting patient's health, safety, and rights.

## Introduction

This report examines a young adult female presenting with third-degree heart block and psychosis, highlighting the ethical and clinical challenges in involuntary hospitalization decisions. We propose a decision-making framework balancing patient autonomy and safety. Upon initial presentation of first-break psychosis (the first onset of psychotic symptoms, such as hallucinations, delusions, and disorganized thoughts), clinical decision-making can be challenging. Shared decision-making frameworks must be established with patients and their support systems. Guidelines are in place to assist clinicians in determining whether a patient requires immediate hospitalization; however, these guidelines have deficiencies that can make hospitalization determinations difficult in complex cases (such as explicitly requiring the expression of homicidal or suicidal ideation) [1]. In our report, we discuss the case of a patient presenting with signs of first-break psychosis who does not fit neatly into involuntary hospitalization (the practice of admitting a patient for psychiatric care without their consent) guidelines.

Patients with mild psychotic symptoms may be able to live safe, independent, and successful lives without ever requiring formal treatment or diagnosis. Often, patients may present to medical attention for unrelated concerns, at which time underlying psychosis may be discovered. Some patients living with psychosis may have mild negative symptoms, “benign” hallucinations, or “quirky” delusions. Additionally, patients may have extensive family and social support during childhood and adulthood, allowing them to remain in the community safely. This support can take on the form of economic support and social acceptance, manifesting as “odd” or “quirky” behaviors. Patients with more mild psychotic features or long-standing features that have ample family support may not clearly alarm others that there has been a significant departure in symptoms that warrant hospitalization.

First-break psychosis often leads to hospitalization due to the out-of-character behaviors of the individual, prompting fear from acquaintances [2,3]. Patients who do not require treatment following first-break psychosis often find themselves hospitalized for treatment or seek medical attention for the treatment of their disorders later in life [4]. Barring the presence of clearly delineated features that fit neatly into the criteria for involuntary hospitalization, physicians can struggle with clinical decision-making when they do not feel confident it is ethical to involuntarily hospitalize the patient based on accepted guidelines [5,6].

In our report, we describe the case of a young adult female who presented to our institution with familial concerns about psychotic behavior and third-degree heart block. The inpatient psychiatric consultation and liaison service struggled with the issue of whether the patient met the criteria for involuntary hospitalization due to the criteria's lack of nuanced language. We hope to present a framework to support psychiatric providers who encounter this common and unsettling patient circumstance with the patient’s well-being and autonomy at the forefront.

## Case presentation

This report addresses the clinical and ethical complexities of involuntary hospitalization in patients with atypical presentations of first-break psychosis, emphasizing the limitations of existing guidelines. This is a young White female, currently domiciled at home with her parent, and unemployed, with a past psychiatric history of presumed bipolar disorder (including depressive episodes), anxiety, and cannabis use disorder, with no history of inpatient psychiatric admissions or suicide attempts, or current psychotropic medication use. She was admitted to the cardiac acute care unit for third-degree heart block. The patient's medical history is otherwise unremarkable. She did not complete high school, struggled to maintain long-term employment, and is financially dependent on her parents. Concerns were raised regarding her parents' opposition to psychopharmacological treatment and their provision of cannabis to “calm her down.”

The patient’s parent reported that the patient was diagnosed with bipolar disorder as a teenager but was never compliant with medication treatment. Her parent reported that over the past few years, the patient exhibited behavioral changes following the death of a family member. Six months before the presentation, the patient’s partner ended their relationship, after which the patient’s behavioral changes worsened, particularly in the two weeks leading up to this evaluation. Within the two months before this presentation, the patient started having severe paranoia and delusions (i.e., being the target of curses, spells, and dark forces, being famous and recognized, still in a relationship, and giving birth to a child with this former partner). Her parents grew increasingly concerned and brought the patient to our emergency department for evaluation.

In the emergency department, the patient was diagnosed with third-degree heart block and admitted to the cardiac acute care unit. Given her psychiatric symptoms, heart block, mild rash on her forearm, and recent exposure to wooded areas, Lyme disease was considered as a potential cause (as this constellation of signs and symptoms is a common presentation of Lyme disease). However, Lyme serologies were negative, and the third-degree heart block appeared to be an incidental finding. Additional workups, including lumbar puncture, autoimmune panel, and neuroimaging, were within normal limits. The patient had a urine toxicology positive for cannabis, mild eosinophilia at 3.5 (reference: 0.0-3.0x10³/µL), and transient leukocytosis at 11.0-13.1 (reference: 4.8-10.8x10³/µL). Otherwise, CBC, chemistries, thyroid, hepatic, and viral panels were within normal limits. The patient was started on empiric ceftriaxone. The physical exam was otherwise unremarkable. Additionally, the patient scored 30/30 on a Mini-Mental State Examination (MMSE) and did not demonstrate signs or symptoms of delirium.

Throughout her hospitalization, the patient exhibited persistent delusions and hallucinations. She had not been sleeping, was poorly compliant with antipsychotic treatment, and lacked any insight into her psychiatric conditions or the seriousness of her cardiac condition. However, the patient consistently denied suicidal or homicidal ideations, depressive thoughts, or command hallucinations. There were also concerns that the patient would not follow up with the cardiology team outpatient for pacemaker placement.

The psychiatric team faced a dilemma as to whether the patient required inpatient psychiatric hospitalization or whether she could be discharged home to her parents. Though the patient appeared “pleasantly psychotic,” she lacked the capacity to leave against medical advice and lacked any insight into her condition. There became a question of whether the patient posed a danger to herself and others despite not reporting suicidal or homicidal ideations. Later in the hospitalization, it became clear that the patient began “catching on” to the concerns of the inpatient teams and began decreasing her reports of her delusions and hallucinations and attempted to linearize her speech. After pressure from the primary cardiac team, the patient was ultimately discharged home with her family and was set up for psychiatric and cardiac follow-up and community psychiatric outreach support from our institution. About three weeks following discharge, the patient was admitted to the inpatient psychiatric unit at our institution for acute and severe psychosis and, to date, has not received the recommended pacemaker.

## Discussion

Ethical considerations

First-break psychosis can present with a broad combination of features, such as hallucinations, delusions, disorganized speech, and negative symptoms [7]. Patient presentation typically informs clinical decision-making, including treatment and discharge disposition plans. Under the New York State (NYS) Mental Hygiene Law, patients can be admitted for psychiatric illness under certain conditions: voluntary, informal, emergency, and involuntary [8]. For involuntary hospitalizations, providers can use the two-physician certificate [8]. The two-physician certificate indicates that “a person has a mental illness for which care and treatment in a mental hospital is essential to his/her welfare; person’s judgment is too impaired for them to understand the need for such care and treatment; as a result of their mental illness, the person poses a substantial threat of harm to self or others" [8]. This allows a person to be held involuntarily for up to 60 days, after which reassessment is required [8]. Initially, two medical doctors must evaluate the patient [8]. Before admission, a staff psychiatrist, besides one of the two original certifying MDs, must confirm that the person meets the involuntary standard [8].

For the other involuntary option, a designee applies, indicating “reasonable cause to believe that the person has a mental illness for which immediate observation, care, and treatment in a hospital is appropriate and which is likely to result in serious harm to themself or others" [8]. "Likelihood of serious harm" means a substantial risk of physical harm to the person, evidenced by threats or attempts at suicide or serious bodily harm, or a substantial risk of physical harm to others, evidenced by homicidal or violent behavior [8]. A medical doctor must then confirm that the person meets the emergency standard, allowing the patient to be held for up to 15 days [8]. Further, NYS Mental Hygiene Law Emergency Standard §9.39 holds that individuals may be admitted for emergency hospitalization if they pose a danger to themselves or others or cannot care for their basic needs, putting their health and safety at significant risk. Involuntary hospitalization can then be extended through a court order [9].

These criteria mainly revolve around harm to self and harm to others. In the absence of these, determining the inability to satisfy basic needs for nourishment, personal care, shelter, or self-protection and suffering worsening mental deterioration and functioning can be more complex. Therefore, evaluating psychiatrists must be particularly exhaustive in obtaining collateral information.

Clinical decision-making challenges

In our patient, the standards above may not apply, given that she did not specifically verbalize a substantial threat of harm to herself or others. With her family's financial and social support, she was questionably able to take care of herself. Though it may be likely that she may have become a danger to herself should she lose the support of her parents, it was unclear whether the involuntary standard or the emergency standard was met. This was further complicated by the challenging family dynamic. Recent studies have shown that patients with more social support are less likely to be involuntarily admitted [10]. The ambiguity of her symptoms was also not apparent at first, leading to the question of whether this unrelated admission required further psychiatric intervention once medically stable. The constellation of cardiac symptoms, time outdoors, and psychiatric complaints initially confused the differential, though ultimately, the third-degree heart block appeared to be a "red herring." Therefore, teams must be cautious of anchoring bias. Further complicating the presentation, the patient lacked any insight into her condition and seemed unwilling to seek follow-up cardiac or psychiatric care; however, the patient’s delivery came off more as a “stubborn” or “bothered” young adult who was not interested in listening to guidance from others. The psychiatric team had concerns that the patient would rapidly decompensate into a dangerous state upon discharge, which ultimately occurred in the weeks following discharge. Since the patient was admitted to the cardiac acute care unit, which is a rapid decision-making unit for discharge or admission, the primary cardiac team encouraged a more expeditious discharge timeline than might have been in the patient's best interest from a psychiatric perspective.

Proposed framework

An unbiased approach must be taken during disposition conversations, with the rationale given for decision-making to grow a trusting patient-physician therapeutic alliance [11,12]. Patient and family views and values must also be taken into account in making shared decisions with an emphasis on compassion, care, understanding, and patient safety. Efforts must be taken to sensitively educate patients and families about recognizing the signs and symptoms of psychosis, the multitude of treatment options, and the likely disease progression. With no risk of harm to self or others, a productive conversation can be had with a compassionate and team approach to the illness. However, sometimes, laws that require explicit phrases such as “dangerous to self or others” as criteria are too rigid and can be dangerous to patients and others [13]. Laws differ from state to state and are constantly evolving, and clinicians must familiarize themselves with the specific language in their practice area. Regardless, the definition of danger to oneself and others can be ambiguous, and interpretation of the law may be outside the scope of practicing physicians. One avenue to pursue is to lean into whether a patient can truly care for themselves or participate in the community and whether they have any insight into their condition to assess the danger posed to themselves. This assessment needs to be made independent of consideration of social and familial support. And therefore, in this case, the patient likely should have been admitted for further psychiatric evaluation and treatment.

## Conclusions

In summary, this is a case of a young adult female patient presenting with likely first-break psychosis and third-degree heart block. The patient appeared to pose no “clear and present danger” to themselves or others. However, she lacked insight into her condition and could not participate in the community. The patient's cardiac condition turned out to be an incidental finding unrelated to her psychiatric symptoms, highlighting the importance of being aware of anchoring bias. The patient was ultimately discharged from acute cardiac care but was admitted to inpatient psychiatric care at our institution three weeks later for florid psychosis and had not followed up with cardiology. This common ethical scenario is challenging for consulting psychiatric services to manage, especially when navigating specialty teams with different clinical and operational focuses. Though ethical guidelines suggest erring on the side of autonomy, one possible approach to ensuring the health and safety of patients and the community in this situation could be to seek involuntary admission because of a lack of insight and inability to participate in the community as a risk to the safety of self. In retrospect, this patient likely met the criteria for inpatient psychiatric admission, which could have sped up her recovery. This case underscores the need for nuanced guidelines that integrate ethical considerations into clinical decision-making for atypical psychosis presentations.
